# pH-Responsive Particle-Liquid Aggregates—Electrostatic Formation Kinetics

**DOI:** 10.3389/fchem.2018.00215

**Published:** 2018-06-14

**Authors:** Peter M. Ireland, Kohei Kido, Grant B. Webber, Syuji Fujii, Erica J. Wanless

**Affiliations:** ^1^Priority Research Centre for Advanced Particle Processing and Transport, University of Newcastle, Callaghan, NSW, Australia; ^2^Division of Applied Chemistry, Graduate School of Engineering, Osaka Institute of Technology, Osaka, Japan; ^3^Department of Applied Chemistry, Faculty of Engineering, Osaka Institute of Technology, Osaka, Japan; ^4^Nanomaterials Microdevices Research Center, Osaka Institute of Technology, Osaka, Japan

**Keywords:** liquid marble, pH-responsive particle, electrostatics, air-water interface, adsorption

## Abstract

Liquid-particle aggregates were formed electrostatically using pH-responsive poly[2-(diethylamino)ethyl methacrylate] (PDEA)-coated polystyrene particles. This novel non-contact electrostatic method has been used to assess the particle stimulus-responsive wettability in detail. Video footage and fractal analysis were used in conjunction with a two-stage model to characterize the kinetics of transfer of particles to a water droplet surface, and internalization of particles by the droplet. While no stable liquid marbles were formed, metastable marbles were manufactured, whose duration of stability depended strongly on drop pH. Both transfer and internalization were markedly faster for droplets at low pH, where the particles were expected to be hydrophilic, than at high pH where they were expected to be hydrophobic. Increasing the driving electrical potential produced greater transfer and internalization times. Possible reasons for this are discussed.

## Introduction

Our group first demonstrated the formation of liquid-particle agglomerates via an electrostatically-driven process in 2013 (Liyanaarachchi et al., 2013). A liquid droplet was produced at the end of an electrically grounded stainless steel capillary above a bed of particles, resting on a substrate to which a negative potential of several kilovolts was applied, causing the particles to jump from the bed to the pendent water droplet. These initial experiments produced metastable agglomerates consisting of a water drop filled with (hydrophilic) glass beads. The new electrostatic process was soon extended to hydrophobic particles (Ireland et al., 2016), which remained embedded at the air-liquid interface instead of entering the droplet. These were genuine “liquid marbles”—liquid drops encased in a shell of non-wetting particles—of the type first observed well over a decade ago (Aussillous and Quéré, 2001), whose remarkable physical properties (Aussillous and Quéré, 2006; McHale and Newton, 2015) rapidly recommended them for a variety of potential applications (Bormashenko, 2017; Oliveira et al., 2017), including gas sensors (Tian et al., 2010), bioreactors (Arbatan et al., 2012a,b), and encapsulation media (Eshtiaghi et al., 2010; Ueno et al., 2014), pressure-sensitive adhesives (Fujii et al., 2016a) and materials delivery carriers (Paven et al., 2016; Kawashima et al., 2017).

The conventional method of forming liquid marbles consists of rolling a droplet on a powder bed. This direct contact method cannot be used to form metastable hydrophilic particle-liquid aggregates, since the liquid would simply soak into a bed incorporating hydrophilic particles. When applied to hydrophobic particles, the new electrostatic method, which did not involve direct contact, was able to circumvent some of the physical limitations of direct-contact formation—for example, larger ratios of particle to drop size were achieved than has previously been considered possible (Eshtiaghi and Hapgood, 2012; Ireland et al., 2016). The electrostatic method has in addition been used to manufacture a new class of liquid marble complexes that include both hydrophobic and hydrophilic particles, resulting in core-shell structures, for a variety of applications, e.g., delivery and controlled release of pharmaceutical powders and water-efficient washing/collection of powder contaminants (Jarrett et al., 2016). Manufacture of even more complex layered structures may also be feasible.

In this context, stimulus responsive materials, whose wettability can be “switched” by various external stimuli (pH, light, temperature, etc.) take on a special significance (Fujii et al., 2016b), as they have the potential to provide even more control over the formation of structured liquid marble complexes. One could envisage a pH-responsive powder being used to safely transfer a liquid through a given environment at high pH, to be released when it reaches a low-pH environment. The utility of these types of mechanisms in drug delivery, for example, is clear. A number of studies have investigated the formation and stability of liquid marbles incorporating stimulus-responsive materials (Fujii et al., 2011; Nakai et al., 2013; Yusa et al., 2014; Paven et al., 2016). In these cases, the marbles were formed by the conventional direct-contact method. Our group has recently attempted to transport stimulus-responsive particles to pendent water droplets by means of the electrostatic method described above in order to assess liquid marble or dispersion formation. The first stimulus-responsive material explored was polystyrene (PS) particles carrying pH-responsive poly[2-(diethylamino)ethyl methacrylate] (PDEA) colloidal stabilizer on their surfaces. PDEA is a weak polybase with a p*K*_a_ of 7.3 that is soluble in aqueous media below pH ~7 because of protonation of its tertiary amine groups (Bütün et al., 2001). At pH 8 or above, PDEA exhibits either very low or zero charge density, and hydrophobic character. It was confirmed that the powders obtained from pH 3.0 and pH 10.0 aqueous dispersions had hydrophilic and hydrophobic characters, respectively. The PDEA-PS powders prepared from pH 3.0 dispersions jumped to a pendent distilled water droplet to form an aqueous dispersion droplet. Conversely, the powders prepared from pH 10.0 did not transfer resulting in no liquid marble formation which was attributed to the cohesive PDEA colloidal stabilizer which resulted in larger grains. This technique may therefore immediately provide a means of preparatively sorting PDEA-PS powders obtained from aqueous dispersions at different pH. A more detailed account of these behaviors, and a comparison between direct-contact (rolling) and electrostatic formation of PDEA-PS marbles, is provided in a recent article by our group (Kido et al., 2018).

One key advantage of the electrostatic aggregate formation technique is the ability to control the final product by changing the kinetics of the formation process via adjustments to the driving potential, drop-bed separation, and bed shape and structure. A pH-responsive particle provides an additional tool for controlling the electrostatic formation kinetics, as it allows the rate at which the particles penetrate the air-water interface to be altered. This paper thus investigates the kinetics of particle transfer and internalization by the droplet of PDEA-coated polystyrene (PDEA-PS) particles during electrostatic formation. Here we focus on PDEA-PS particles that were dried from a solution at pH 3, as these were consistently transported to the pendent droplet.

## Methodology

### Particle synthesis and experimental method

The PDEA-PS particles were synthesized by dispersion polymerization using PDEA homopolymer as a colloidal stabilizer in the same way reported previously (Sekido et al., 2017). The PDEA-PS particles were nearly monodisperse and had a number-average diameter of 2.20 μm and a coefficient of variation of 2%. PDEA loading was determined to be 2.66 wt% by elemental microanalysis and XPS studies on the PDEA-PS particles determined the surface coverage by PDEA to be approximately 47%. These results indicate that the PDEA is mainly located at the surface of the PS particles. Aqueous dispersion of PDEA-PS particles with a pH value of 3.0 (40 g, 10 wt%, adjusted using HCl aqueous solution) was dried at 21°C, 0.1 MPa and 42.8–87.4 RH% in air. The obtained dried cake-like white agglomerate was ground into powder using a pestle and mortar.

**Figure 1:**
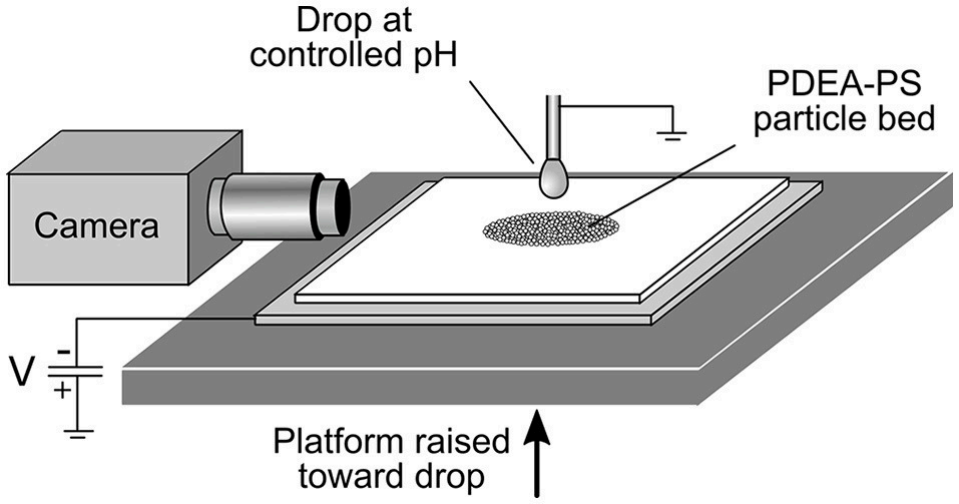
Schematic of the apparatus used for electrostatic formation of liquid marbles.

A schematic of the experimental apparatus is shown in Figure 1. A bed of dried PDEA-PS particle powder, depth ~1 mm, was supported by a 1 mm thick glass slide, which in turn rested on a stainless steel plate connected to a high voltage power supply. The metal plate was held at a constant negative potential relative to earth of 1.5, 2.0, or 2.5 kV, and was gradually raised at a rate of 50 μm·s^−1^ toward a pendent drop on the end of an earthed metal capillary syringe of 1.2 mm outer diameter. The drop liquid was either MilliQ water (pH 5.6), or an aqueous buffer at pH 3.0 (0.1 M potassium hydrogen phthalate/HCl) or 10 (NaHCO_3_/NaOH) (De Lloyd, 2000). The nominal drop volume was 5 μL, as dispensed by a syringe pump (Harvard Apparatus 11Plus), before the application of the electric field and loading with particles. When the separation between the particle bed and drop became sufficiently small (between ~0.7 mm for 1.5 kV and ~2.0 mm for 2.5 kV), the powder was transferred across the gap to the drop, before becoming internalized within the drop. A video camera was used to record the entire process from the start of particle transfer to the completion of particle internalization.

### Preliminary observations and hypotheses

**Figure 2:**
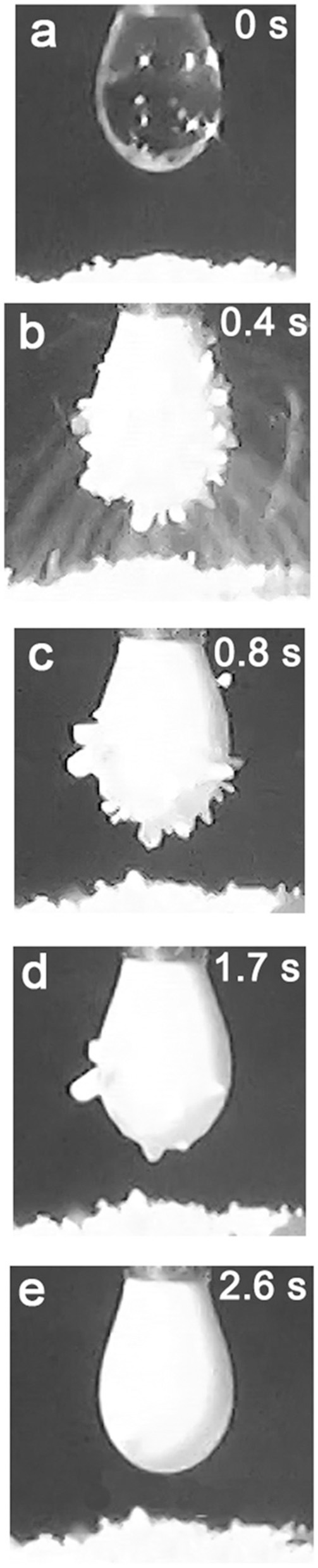
Electrostatic transfer, followed by internalization, of PDEA-PS powder (dried from a solution at pH 3) into a water droplet, for a pH 5.6 droplet and a driving potential of 2.5 kV.

Preliminary electrostatic aggregation experiments with PDEA-PS particles revealed several interesting behaviors. The powder tended to jump to the drop not as individual particles, but as grains of substantial size containing many individual particles (Figure 2). For more information on the granular properties of this material, please refer to Kido et al. (2018). These initially attached to the air-water interface, before gradually being internalized by the droplet. Thus, the initially-smooth drop surface (a) took on a “jagged” appearance as it was encrusted with PDEA-PS grains (b), then gradually became smooth again once particle/grain transfer ceased (c–e). This cessation normally corresponded to depletion of that part of the particle bed that was subject to transfer to the drop, i.e., the section of the bed where the electrostatic force was sufficient to counterbalance gravity and cohesive or static friction forces (Ireland et al., 2015). Since the particles were all eventually internalized by the drop, no stable liquid marbles were formed. It can be argued, however, that metastable liquid marbles were manufactured. These might be viewed as exhibiting “delayed instability”—starting as fully-stable liquid marbles, but losing that stability at a rate that can in principle be controlled by pH. This property may be useful in applications where the rate of release of a liquid into the surrounding environment needs to depend on the pH.

Given these observations, the process is conceptualized in terms of two main kinetic parameters—the time-scales for transfer of grains from the bed to the surface of the drop, and for internalization of grains attached to the air-water interface. The precise mechanism of internalization is not known, and may have included wicking of water into the interstices of the multiparticle PDEA-PS grains, as well as engulfment of the entire grain. The rate of both processes was expected to depend on the wettability of the particle surface. Since the PDEA surface of the particles was hydrophilic at pH values below the p*K*_a_ of 7.3 and hydrophobic at higher pH values, it was hypothesized that the internalization process would be more rapid at low pH values of the drop liquid than at high values. A long internalization time would presumably promote an accumulation of grains at the drop surface, and it was hypothesized that this would suppress transfer and attachment of additional grains to the drop surface. Lower drop pH values were therefore expected to result in more rapid transfer as well as more rapid internalization.

Given the evolution of the droplet silhouette shown in Figure 2, the characteristic internalization and transfer times were estimated from video footage of the process using a two-stage transfer-internalization model and a fractal-based analysis technique. These characteristic times were compared for different values of the driving potential and drop pH, to test the above hypotheses.

### Data analysis

The fractal dimension of each aggregate's silhouette outline, as it evolved over time, was used to characterize the transfer and internalization kinetics. It was selected as an appropriate measure because of its robustness and scale-independence (Rasband, 1990). The fractal dimension gives a measure of the degree of “convolutedness” of the aggregate outline (Falconer, 1990). A drop outline without particles, or with entirely internalized particles, was smooth, with fractal dimension close to 1. The PDEA-PS powder tended to transfer to the drop surface in the form of multiparticle grains rather than individual particles, and there was thus a substantial increase in the fractal dimension of the drop outline when non-wetted grains were present at its surface. The scale-independence of the fractal dimension was considered to more than compensate for the fact that there was no obvious exact correlation between the fractal dimension and the number of particles at the bubble surface. Since we were chiefly interested in the rate constants for the process, not the actual numbers of particles, this was a secondary consideration. The fractal dimension was calculated using a standard box-counting algorithm, details of which are provided in the Supporting Information.

### Model

To assist in interpretation of kinetic data, a simple model of the transfer to and internalization of particles by the drop was developed. The drop surface is conceptualized as possessing a number of sites, *N*_*d*_, to which particles (or in this case, grains) can attach. These sites can either be directly on the air-liquid interface, or may represent adhesion of incoming particles/grains to other particles/grains. The section of the particle bed beneath the drop possesses *N*_*b*_ such sites. We let *n*_*d*_ and *n*_*b*_ be respectively the number of occupied sites on the drop surface and bed. It is assumed that the rate of change of the number of particles or grains in the bed is equal and opposite to the rate at which they jump to the drop, and that this is proportional to *n*_*b*_. It is further assumed that only those particles/grains that jump to an unoccupied site on the drop surface are able to attach. The probability of this is proportional to the fraction of sites on the drop surface that are unoccupied, i.e., 1−*n*_*d*_/*N*_*d*_ . Thus

(1) $$\frac{dn_{b}}{dt}=\;-\left(Transfer\;rate\right)=-\frac{1}{\tau_{t}}n_{b}\left(1-\frac{n_{d}}{N_{d}}\right)$$

where τ_*t*_ is a time constant for particle transfer. In addition to this transfer process, which fills sites on the drop surface, particles/grains enter the interior of the drop, resulting in formerly occupied sites at the drop surface becoming unoccupied. The internalization rate is assumed to be proportional to the number of occupied states at the drop. Thus, the rate of change of the number of occupied sites on the drop surface is given by

(2) $$\frac{dn_{d}}{dt}\;=\left(Transfer\;rate\right)-\left(Internalisation\;rate\right)\;=\frac{1}{\tau_{t}}n_{b}\left(1-\frac{n_{d}}{N_{d}}\right)-\frac{1}{\tau_{i}}n_{d}$$

where τ_*i*_ is the time constant for the internalization process. Since we are not able to measure absolute values of *n*_*b*_, *n*_*d*_, *N*_*b*_, or *N*_*d*_, but only relative values, we can reduce the number of model variables by introducing the following ratios:

(3) $$\nu =\frac{n_{b}}{N_{b}}$$

(4) $$\mu =\frac{n_{d}}{N_{d}}$$

(5) $$\eta =\frac{N_{b}}{N_{d}}$$

in terms of which Equations (1) and (2) take the simpler forms

(6) $$\frac{d\nu }{dt}=-\frac{1}{\tau_{t}}\nu \left(1-\mu \right)$$

(7) $$\frac{d\mu }{dt}=\frac{1}{\tau_{t}}\eta \nu \left(1-\mu \right)-\frac{1}{\tau_{i}}\mu$$

with only three instead of four parameters. All of the sites on the bed are initially occupied, and those on the drop surface are initially unoccupied, so

(8) ν(t=0)= 1; μ(t=0)= 0

When ν is reduced to zero, the region of the bed from which particles are able to jump is depleted (we see this physically as a region of bare substrate underneath the drop or aggregate).

## Results and discussion

**Figure 3:**
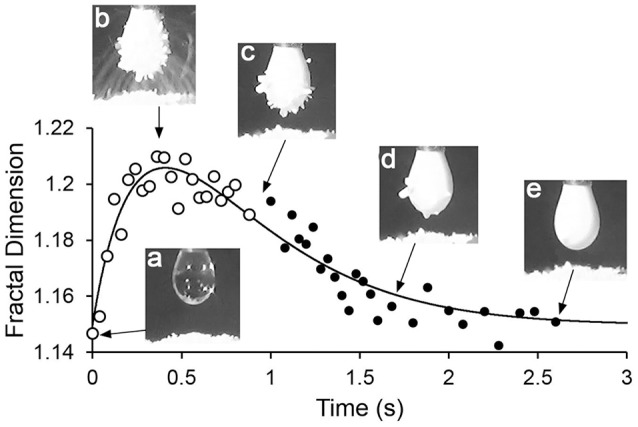
Plot of fractal dimension of the aggregate outline during particle transfer and internalization, with drop pH 5.6 and driving potential 2.5 kV.

Figure 3 shows a plot of fractal dimension of the aggregate outline as a function of time for the transfer-internalization process of PDEA-PS particles to a droplet of pH 5.6 water under a 2.5 kV applied voltage. The increase in the fractal dimension during particle transfer and subsequent decrease with internalization is clearly apparent. Note that the fractal dimension is between 1.1 and 1.3, as we would expect for an outline that is somewhat convoluted in two dimensions, but is not near being space-filling.

### Extraction of parameters

The fractal dimension vs. time data (Figure 3) were now fitted to Equations (6) and (7). Since, as already mentioned, there was no clear correlation between μ and the fractal dimension *d*, a linear correlation of the form

(9) d(μ)=Aμ+B

was used, where *A* and *B* are auxiliary constants corresponding to the proportionality of μ and *d* and the fractal dimension of the smooth drop outline, respectively. It was discovered that a fit in which τ_*t*_, τ_*i*_, η and the two auxiliary constants were adjusted simultaneously could produce one or more spurious solutions. This was prevented by first determining τ_*i*_, *A* and *B*. The time at which grains ceased jumping from the bed to the drop was estimated by inspecting the video footage. With no particle transfer, both sides of Equation (6) and the first term on the right-hand side of Equation (7) become zero, and the solution of (7) is simply an exponential decay with time constant τ_*i*_. A curve of this form was fitted to the data after cessation of transfer (the section of the data in Figure 3 after c, ~0.9 s) using total least squares regression, with τ_*i*_, *A* and *B* as adjustable parameters, weighted equally for variation along the time and fractal dimension axes. Once τ_*i*_ was determined, τ_*t*_ and η were found using a total least-squares fit of a numerical solution of Equations (6) and (7) to all the data. Uncertainties in the parameters were determined at a confidence level of 95% using 400 Monte Carlo simulations per fit, as in the method of Hu et al. (Hu et al., 2015). The uncertainty in both time constants was of the order of 0.1 s, and that of the parameter η was also ~0.1. The parameter *A* ranged from 0.03–0.037 for 1.5 kV experiments, 0.03–0.046 for 2.0 kV experiments, and 0.06–0.1 for 2.5 kV experiments, reflecting the different coverage patterns observed at different driving potentials. *B*, the ‘baseline' fractal dimension for a bare drop, varied slightly from ~1.135 at 1.5 kV to 1.15 at 2.5 kV, probably due to greater electrostatic elongation of the drop at higher driving potentials.

### Kinetics

**Figure 4:**
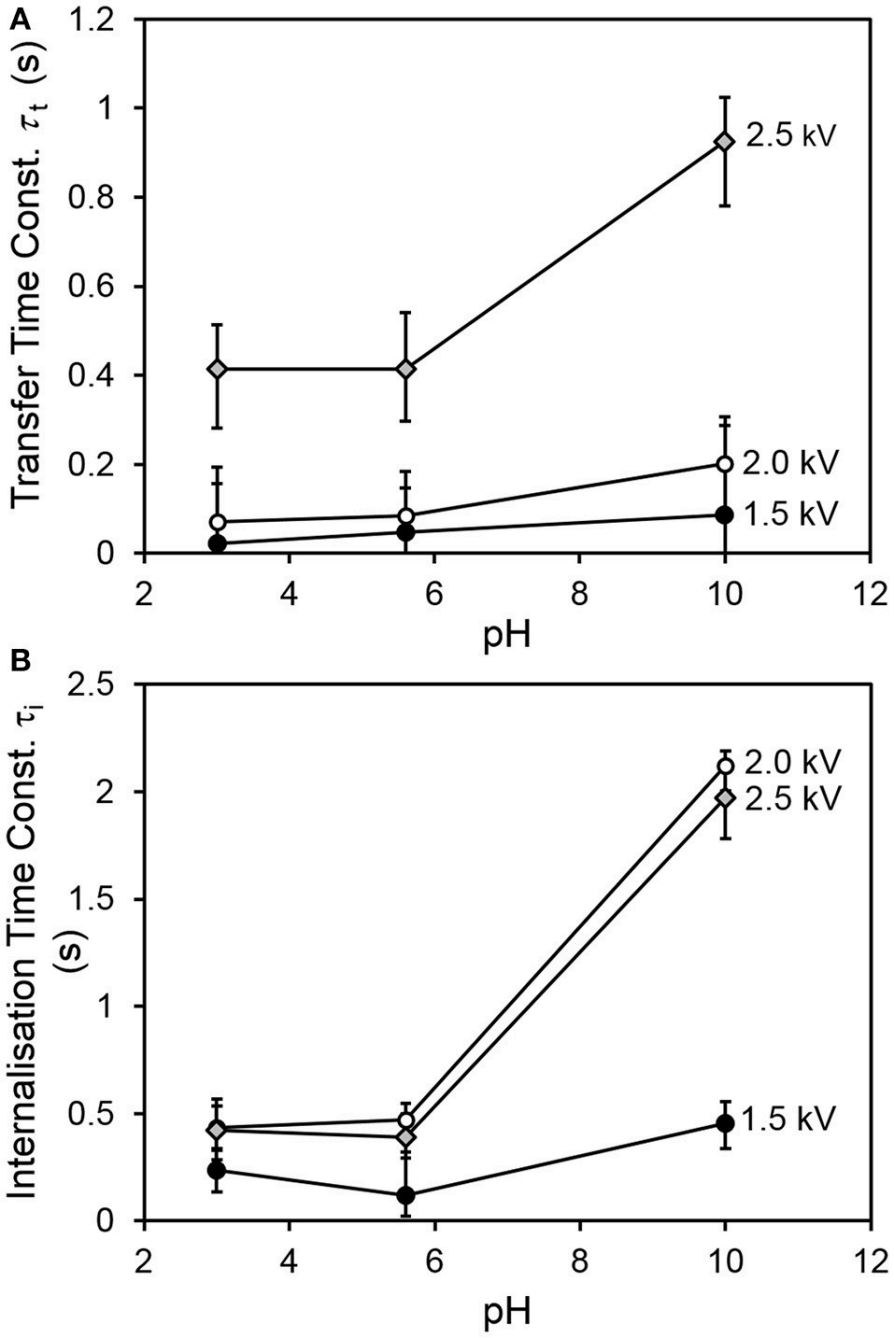
**(A)** Characteristic transfer time and **(B)** characteristic internalization time vs. pH for all values of driving potential.

Figure 4 shows the transfer and internalization time constants respectively as a function of the driving potential and the drop pH. It is clear from Figure 4A that the transfer process is slower for a drop pH of 10, compared to drops at lower pH values. Figure 4B indicates that the internalization time follows a similar trend with drop pH. The latter trend is consistent with our expectations—since the p*K*_a_ of PDEA is 7.3, we would expect the particles to be wettable at pH 3 and 5.6, and non-wettable at pH 10. The increase in transfer time with drop pH was also expected, as slower internalization means that transferred particles vacate drop surface states more slowly, and it was hypothesized that the rate of transfer would have an inverse relationship to the extent of occupation of the drop surface (recall Equations 6 and 7). Two different mechanisms are proposed for this hypothesized relationship. The first involves physical obstruction: a grain jumps from the bed but encounters a part of the drop surface already occupied by a non-wetted grain, and simply bounces off rather than attaching to the air-water interface. The second proposed mechanism involves an accumulation of net negative charge at the drop surface due to the presence of non-wetted charged grains, which have made only partial contact with the water and thus have not been fully neutralized. This accumulation of net negative charge at the drop surface would be expected in turn to decrease the electric field between the drop and the bed.

The increase of internalization time with driving potential initially seems counter-intuitive. It is hypothesized that at larger driving potentials, multiple layers of grains are able to adhere to the drop. Only a fraction of the adherent grains (the innermost) is in direct contact with the liquid at any given time. In our simple model, the internalization rate is proportional to the number of occupied sites at the drop surface. No distinction is made between the time taken for grains in contact with the liquid to be internalized, and the time spent by adherent grains in the outer parts of the multilayer, waiting for inner grains to be internalized by the drop before making contact with the liquid themselves. The modeled internalization time is actually the sum of these two distinct processes, and will thus tend to be longer for multilayer coverage (as at higher driving potentials).

**Figure 5:**
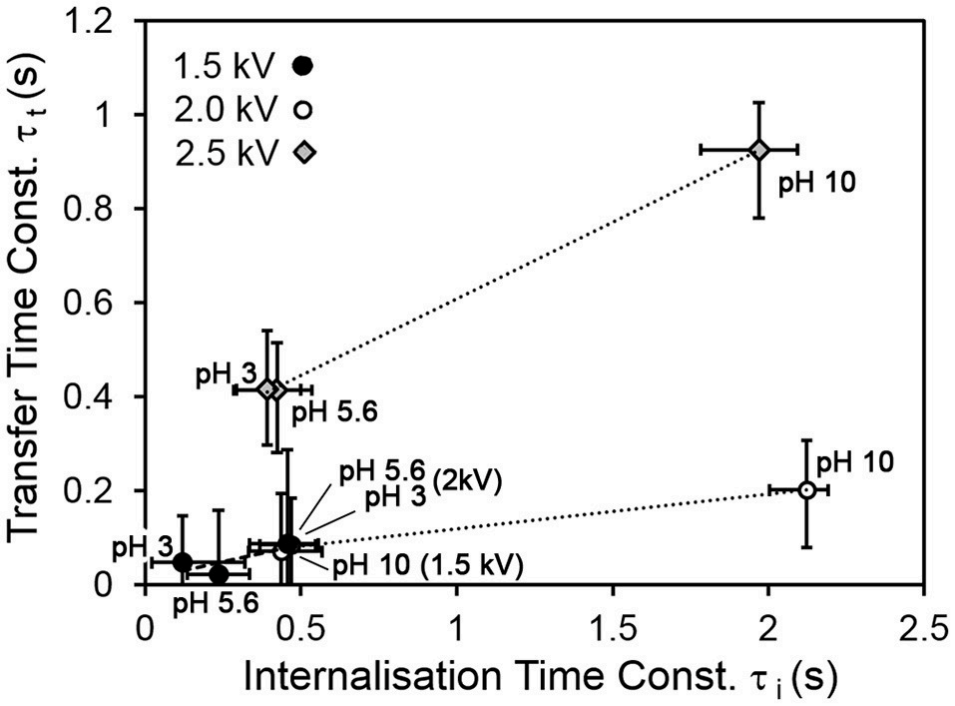
Transfer time vs. internalization time for all pH and driving potential values.

**Figure 6:**
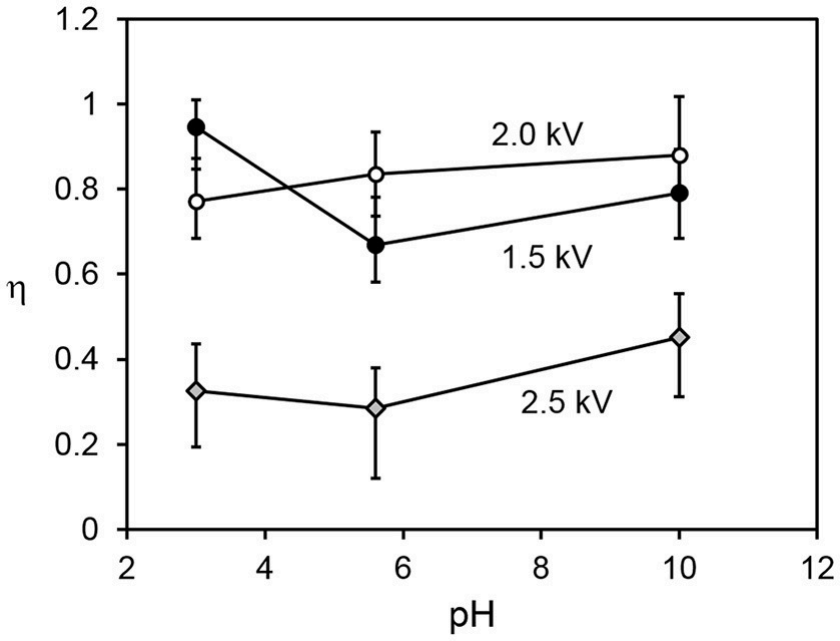
Ratio of total number of available sites in the particle bed to those at the drop surface vs. pH, for all driving potential values.

Figure 5 shows the relationship between the transfer and internalization time constants, for all driving potentials and pH values. The 2.5 kV experiments exhibit a larger transfer time constant for the same internalization time constant than those at lower driving potentials. The reasons for this are not yet clear. A clue may be provided by Figure 6, which shows the parameter η (recall Equation 5), representing the ratio of the total number of available sites for grains in the bed to that on the drop surface, as a function of pH. This suggests that in the 2.5 kV case, the number of available sites on the drop surface was relatively large for the number of grains able to jump to them, compared to the lower-potential cases, between which there is no significant difference. It seems reasonable that an increasing driving potential would increase the total number of available sites in both the bed and at the drop, but it is not clear why it would preferentially increase the number at the drop compared to those in the bed. This may be related to the shape of the electric field. For a larger driving potential, the electrostatic force is able to overcome cohesive forces in the bed and the particle/grain weight at a greater drop-bed separation. Thus, the geometry of the system is different during transfer at different driving potentials. On the other hand, pH appears to have only a weak effect, if any, on η. This is consistent with our model, which assumes that pH influences the rate at which occupied sites become vacant by internalization, not the actual number of those sites.

**Figure 7:**
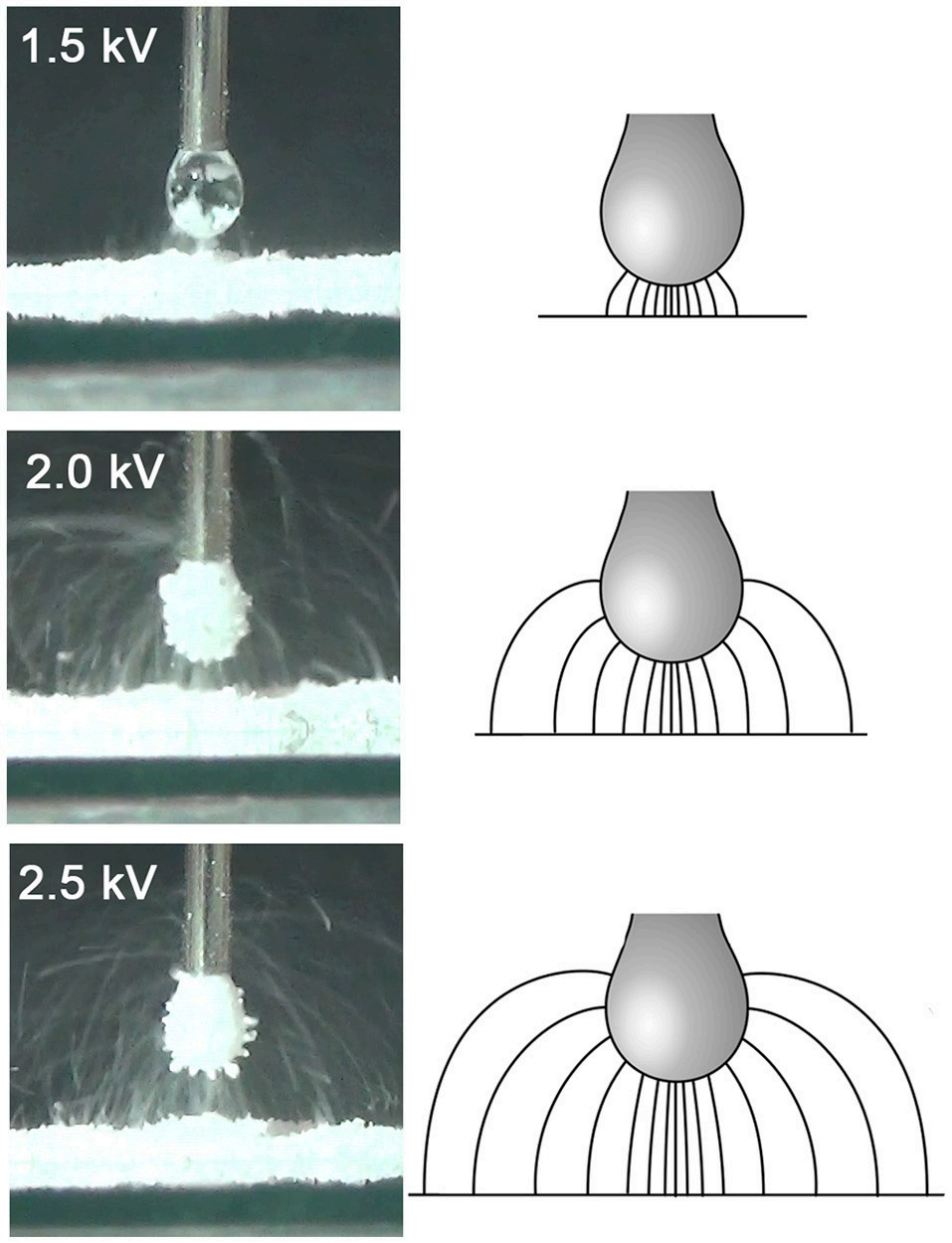
Schematic of electric field lines and photographs of particle trajectories for equivalent stages in the particle transfer process (at approximately the time of the peak fractal dimension) at different values of the driving potential (and hence drop-bed separation). The photographs are all for a drop at pH 5.6.

Figure 7 shows a schematic of the electric field lines, and photographs of the corresponding stage of particle transfer, for a droplet at pH 5.6 and all three driving potentials (note that due to inertia and gravity, the particle trajectories differ somewhat from the field lines). For aggregate formation at lower driving potential values, the field lines and particle trajectories are concentrated under the lower part of the droplet, and originate from a much smaller area of the bed, with relatively direct particle trajectories. At higher driving potential values, the grains come from a much larger area of the bed and are transferred to the entire surface of the droplet, often via long, curving trajectories. It is hypothesized that this change in the morphology of the grain trajectories results in a larger relative increase in the number of available sites on the drop surface than it does the number of grains in the bed able to jump. Assessment of this hypothesis will require numerical modeling of the field shape and particle trajectories. We plan to explore the relationship between field morphology and the transfer kinetics in more detail in subsequent studies, using the COMSOL Multiphysics finite-element modeling environment (COMSOL Inc., Burlington, MA).

## Conclusions

Our group's novel electrostatic method for manufacturing liquid marbles and other types of liquid-particle agglomerates was applied to a stimulus-responsive material, polystyrene particles coated in pH-responsive poly[2-(diethylamino)ethyl methacrylate] (PDEA), whose surface was hydrophilic at low pH and hydrophobic at high pH. The formation of aggregates with these PDEA-PS particles was modeled as two coupled processes, namely transfer of particles from the bed to the droplet surface, and internalization of the particles into the droplet. Since all particles in these experiments were eventually internalized, stable liquid marbles were not formed—instead, metastable liquid marbles were created. The kinetics of the transfer/internalization process were characterized using fractal analysis of video footage to track the “roughening” and “smoothing” of the droplet silhouette as grains were transferred to it and then internalized. Consistent with expectations, the characteristic time for internalization was found to be markedly more rapid when the pH of the drop was below the p*K*_a_ of PDEA (i.e., when the particle surface was expected to be hydrophilic) than when it was above the p*K*_a_ (when it was hydrophobic). Grain transfer was also found to be more rapid at low pH, since more rapid internalization made more surface sites available for grains to jump to. The variation of transfer and internalization times with driving potential was broadly as expected, although full explanation of some aspects will require detailed modeling of the field morphology.

## Author contributions

KK performed the experiments under supervision of the other authors. PI developed the model and performed the analyses. SF synthesized and characterized the particles. PI, SF, GW, and EW wrote the article.

### Conflict of interest statement

The authors declare that the research was conducted in the absence of any commercial or financial relationships that could be construed as a potential conflict of interest.
