# Supplementary material for: pH-Responsive Particle-Liquid Aggregates—Electrostatic Formation Kinetics
Source: Front Chem. 2018 Jun 14;6:215. doi: 10.3389/fchem.2018.00215 (PMC6010524; doi:10.3389/fchem.2018.00215)
Supplement: Supplementary file 1 [file Image_1.PDF]

Supplementary Material

pH-responsive Particle-Liquid Aggregates – Electrostatic Formation Kinetics

Peter M. Ireland,* **Kohei Kido, Grant B. Webber, Syuji Fujii, Erica J. Wanless**
***Correspondence:** Peter M. Ireland: Peter.Ireland@newcastle.edu.au

The fractal dimension of the aggregate outline was calculated using a standard box-counting algorithm (Falconer, 1990). For each video frame of the formation and internalisation process, an automated Adobe Photoshop routine idenfied the 2D outline of the aggregate (Figure A1). Another automated routine then performed a box-counting procedure on this outline. The original outline image was coarsened by a series of integer factors *s* as shown in Figure A1. (Each coarsening step was applied to the original outline, not the previous coarsened version.) The number of these progressively larger 'boxes' occupied by the aggregate outline, *N*(*s*), was counted for each coarsening factor. Since small changes in the alignment of the box array relative to the image can introduce substantial variability in the box count (Falconer, 1990) five small random transpositions of each image were analysed and averaged to produce the final array *N*(*s*). For an 'ideal' fractal shape, one should find that

$$\log N(s) = d \log s \qquad (S1)$$

where *d* is the fractal dimension. Thus, to estimate *d*, log *N*(*s*) was plotted against log *s*, and a linear regression analysis performed. The slope of this regression line was the fractal dimension of the aggregate outline.

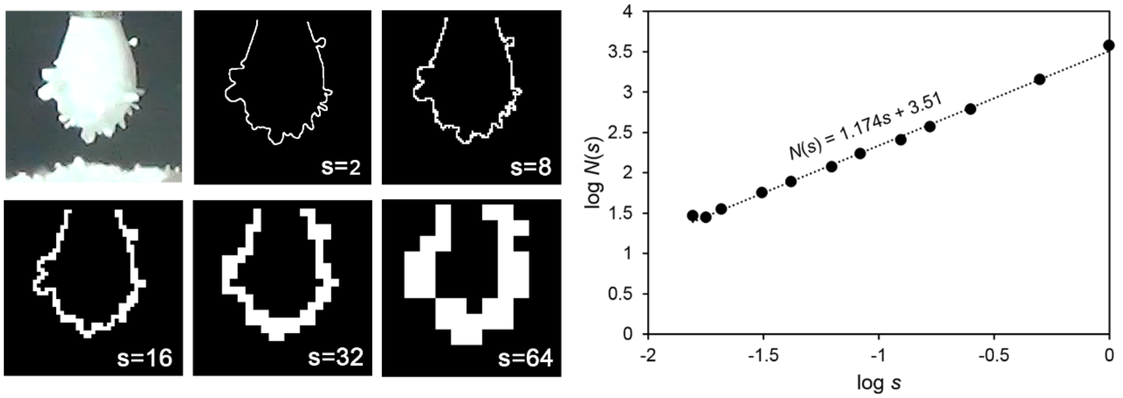

Supplementary Figure 1. Progressively-coarsened versions of the aggregate silhouette used in box-counting, and the resulting fit used to calculate the fractal dimension. Image is for PDEA-PS particles transported to a water droplet at pH 5.6 under a 2.5 kV applied voltage.

References

Falconer, K. (1990). *Fractal geometry: Mathematical foundations and applications.* Chichester: John Wiley.
